# RNA Interference as a Method for Target-Site Screening in the Western Corn Rootworm, *Diabrotica Virgifera Virgifera*

**DOI:** 10.1673/031.010.14122

**Published:** 2010-09-24

**Authors:** Analiza P. Alves, Marcé D. Lorenzen, Richard W. Beeman, John E. Foster, Blair D. Siegfried

**Affiliations:** ^1^ Department of Entomology, University of Nebraska — Lincoln, Lincoln, NE 68583-0816; ^2^ USDA-ARS-CGAHR, 1515 College Ave., Manhattan, KS 66502

**Keywords:** biopesticide, *chitin synthase 2*, *laccase 2*, systematic RNAi

## Abstract

To test the efficacy of RNA interference (RNAi) as a method for target-site screening in *Diabrotica virgifera virgifera* LeConte (Coleptera: Chrysomelidae) larvae, genes were identified and tested for which clear RNAi phenotypes had been identified in the Coleopteran model, *Tribolium castaneum*. Here the cloning of the *D. v. vergifera* orthologs of *laccase 2 (DvvLac2)* and *chitin synthase 2 (DvvCHS2)* is reported. Injection of *DvvLac2*-specific double-stranded RNA resulted in prevention of post-molt cuticular tanning, while injection of *DvvCHS2-*specific dsRNA reduced chitin levels in midguts. Silencing of both *DvvLac2* and *DvvCHS2* was confirmed by RT-PCR and quantitative RT-PCR. As in *T. castaneum*, RNAi-mediated gene silencing is systemic in *Diabrotica*. The results indicate that RNAi-induced silencing of *D. v. vergifera* genes provides a powerful tool for identifying potential insecticide targets.

## Introduction

The western corn rootworm, *Diabrotica virgifera virgifera* LeConte (Coleoptera: Chrysomelidae), is one of the most important insect pests of corn throughout the U.S. Corn Belt (Levine and Oloumi-Sadeghi 1991) both in terms of crop losses and synthetic insecticide use. Crop rotation and chemical control have been the primary management strategies to reduce root damage caused by larval feeding (Levine and Oloumi-Sadeghi 1991). However, the evolution of resistance to different insecticide classes (Ball and Weekman 1962; Meinke et al. 1998; Zhou et al. 2002) and behavioral resistance to crop rotation (O'Neal et al. 2001; Levine et al. 2002) has made rootworm control increasingly difficult. Novel control techniques currently used in rootworm management include transgenic corn hybrids expressing *Bacillus thuringiensis (Bt)* toxins and seed treatment with neonicotinoid insecticides. The adoption of transgenic technologies has been rapid (Pilcher and Rice 1998; James 2002, 2004, 2006; Rice 2003), raising concerns about the further evolution of resistance. The limited number of alternative control strategies and the history of rootworm adaptation call for the discovery of new insecticide targets (Meinke et al. 1998; O'Neal et al. 2001; Levine et al. 2002; Zhou et al. 2002).

The success of transgenic plants expressing *Bt* toxins emphasizes the importance of identifying target sites that provide both selectivity and environmental safety. The rootworm genome offers an important resource for such target-site discovery. An important component of this discovery process will involve validation using functional-genomic techniques such as RNA interference (RNAi). RNAi is a naturally occurring defense mechanism that is triggered by double-stranded RNA (dsRNA) and functions to protect cells from parasitic nucleic acids (Bellés 2010). Briefly, the RNAi process starts by cleavage of dsRNAs into sequence-specific effector molecules (small interfering RNAs or siRNAs) which target homologous RNAs for destruction (Dykxhoorn et al. 2003; Hannon 2002). These siRNA are then incorporated into a multiprotein RNA-inducing silencing complex (RISC) (Nykanen et al. 2001), eventually leading to endonucleolytic cleavage and resulting in silencing of the homologous target mRNA (Elbashir et al. 2001; Hannon 2003). The use of RNAi technology for knockdown of target mRNA has become a powerful tool in studying protein function (Bosher and Labouesse 2000; Dykxhoorn et al. 2003). Delivery of dsRNA either by feeding or injection (Kennerdell and Carthew 1998) provides rapid, but transient degradation of mRNA (Kennerdell and Carthew 2000; Timmons and Fire 1998). Through this reverse genetics approach, a gene is disrupted so that the effect of its loss on an organism can be observed (Waterhouse and Helliwell 2002). Phenotypic consequences of transcript knockdown by RNAi can approximate insecticidal effects on a given target, and the timing and severity of defects caused by RNAi should provide an important indicator of target utility (Behm et al. 2005).

The efficacy of RNAi to silence genes in *D. v. virgifera* was recently documented by Baum et al. (2007), who reported the silencing of the housekeeping genes *vacuolar ATPase A (VATPase A)* and α*-tubulin*. Larval feeding assays documented that corn plants expressing *V-ATPase A* dsRNA caused mortality to *D. v. virgifera* larvae and effectively suppressed feeding damage to root tissue. These results suggest strongly that *D. v. virgifera* is amenable to systemic, RNAi-mediated gene silencing, as previously reported for *Tribolium castaneum* (Tomoyasu et al. 2008).

To test RNAi as a tool for target-site validation in *D. v. vergifera*, orthologs of genes with easily recognized RNAi phenotypes in *T. castaneum* were chosen. The first gene, *lacease 2 (Lac2)*, encodes a phenoloxidase required for larval, pupal, and adult cuticle sclerotization and pigmentation in *T. castaneum* (Arakane et al. 2005a). RNAi-mediated silencing of *T. castaneum Lac2 (TcLac2)* prevents post-molt pigmentation of the integument in this insect. The second gene, *chitin synthase 2 (CHS2)*, belongs to a large family of membrane-bound glycosyl transferases (Cohen 2001; Merzendorfer 2006) and catalyzes the polymerization of N-acetylglucosamine (GlcNAc) monomers into chitin. Arakane et al. (2005b) demonstrated that *TcCHS2* from *T. castaneum* is directly involved in the synthesis of the chitin required for proper formation of the midgut peritrophic matrix. Silencing of *TcCHS2* leads to death by starvation, presumably due to the inability of a chitinless peritrophic matrix to mediate digestion and absorption of dietary nutrients. The current work examines whether similar RNAi phenotypes for *Lac2* and *CHS2* are seen in *D. v. virgifera*.

Here the cloning of the *CHS2* and *Lac2* genes from *D. v. virgifera* (from here on referred to as *DvvCHS2* and *DvvLac2*, respectively) is reported and expression data for two larval instars are provided. The effects of RNAi-mediated gene silencing of these two genes are also reported, and transcript knockdown via both RT-PCR and qRT-PCR is demonstrated. The results indicate that RNAi- induced silencing of *D. v. virgifera* genes provides a powerful tool for identifying and validating potential target sites as well as analysis of gene function in this important pest species.

## Materials and Methods

### Insect strains and rearing

All *D. v. virgifera* larvae used in this study were obtained from a non-diapause colony and purchased from Crop Characteristics, Inc. (www.cropcharacteristics.com). Larvae were maintained at room temperature (approximately 23° C) on roots of germinated corn seeds. After injection, larvae were placed in a Petri dish containing moistened filter paper for observation. Upon recovery from injection trauma, larvae injected with *DvvLac2*-specific dsRNA were transferred to germinated seed corn grown in a gel matrix (Clark 2002). This enabled individual larvae to be observed and examined after molting. Larvae injected with *DvvCHS2*-specific dsRNA were transferred to two-ounce plastic cups containing peat moss (3:1 peat moss:water) and a single corn seedling. Transferred larvae were maintained at 25° C, 12:12 L:D and 40–50% relative humidity.

### Homology-based *D. v. virgifera* gene cloning

**Figure 1.**
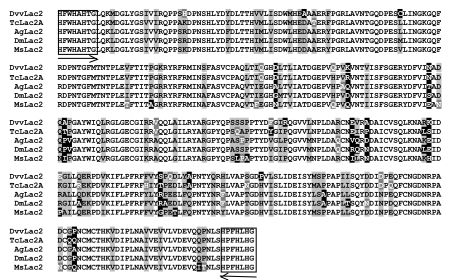
Multiple aligmnent of amino acid sequences of the deduced laccase 2 from *Diabrotica virgifera virgifera* (DvvLac2) with *Tribolium castaneum* (TcLac2a), *Anopheles gambiae* (AgLac2), *Drosphila melanogaster*, and *Manduca sexta* (MsLac2). Boxed areas indicate the sequences used to synthesize degenerate primers for fragment amplification. Arrows indicate direction of amplification. High quality figures are available online.

**Figure 2.**
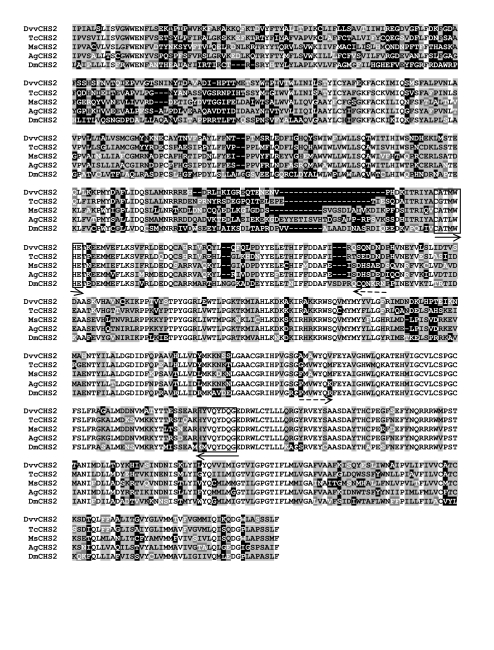
Multiple amino acid alignment of the deduced *Diabrotica virgifera virgifera* chitin synthase 2 (DvvCHS2), with *Tribolium castaneum* (TcCHS2), *M. sexta* (MsCHS2), *Anopheles gambiae* (AgCHS2), and *Drosophila melanogaster* (DmCHS2). Boxed areas indicate sequences used to design degenerate primers for amplification of the initial *DvvCHS2* specific fragment. Dashed arrows indicate primers used for obtaining additional sequence in the 3′- and 5′- directions. High quality figures are available online.

*D. v. virgifera*-specific gene sequences were obtained using a combination of degenerate-PCR with total RNA extracted from whole bodies of third instars reverse transcribed to complementary DNA (cDNA) and RACE (Rapid Amplification of cDNA Ends) Degenerate primers were designed based on conserved regions identified from the alignment of amino-acid sequences from other insect species. For *laccase 2* the alignment included Lac2 sequences from *Anopheles gambiae* (AAX49501), *Drosophila* *melanogaster* (NP_724412), *Manduca sexta* (AAN17507), and *T. castaneum* (AAX84202) (see Figure 1). For *chitin synthase 2* the alignment included *CHS2* sequences from *A. gambiae* (XP_321336), *D. melanogaster* (AAF51798), *M. sexta* (AAX20091), and *T. castaneum* (NP_001034492) (see Figure 2). Degenerate primers were designed in regions having high sequence conservation and low codon degeneracy (Figures 1–2). To obtain transcript specific templates for the desired regions of *Lac2* and *CHS2*, degenerate PCR was performed using cDNA as template. The following primer pairs were used: 5′CAYTTYTGGCAYGCNCAYACNGG-3′ and 5′- CRTGNARRTGRAANGGRTG-3′ for *Lac2A*, which correspond to the highly conserved amino-acid sequence identified in Figure 1; and 5′-TGYCGNACNATGTGGCAYGAR-3′ and 5′-CCYTGRTCRTAYT GNACRTARTG-3′ for *CHS2*, which correspond to the highly conserved amino-acid sequence of *CHS2* CATMWHE and QYDQGED (Figure 2). To obtain *DvvLac2* sequence, degenerate PCR was performed with an initial denaturation cycle of three min at 94° C, followed by 30 cycles of 94° C denaturation for 30 sec, 61.7° C annealing for *DvvCHS2* sequence, degenerate PCR was performed with an initial denaturation cycle of two min at 94° C, followed by 35 cycles of 94° C denaturation for 30 seconds, 51° C annealing for 40 sec, and 72° C extension for three min. Control PCR reactions containing sterile water in the place of template were conducted in both PCR reactions. PCR products were separated on 1% agarose gel in 1 × TBE containing 0.5 µg/ml ethidium bromide and visualized with UV light. *D. v. virgifera*-specific PCR fragments were cloned using the TOPO TA Cloning Kit (pCR2.1-TOPO vector; Invitrogen, www.invitrogen.com) and sequenced at the University of Nebraska Genomics Core Research Facility for template confirmation, and their alignments are shown in Figures 1 and 2.

To amplify a longer *DwCHS2* cDNA and obtain a fragment having higher sequence dissimilarity between *TcCHS1* and *TcCHS2*, both 3′- and 5′-RACE PCR were performed (Figure 2). The primers 5′-TTCAGGTGCAATGGCTTGGTATC-3′ and 5′-CTCGTAGTAATCAGGCAGTTGGAA- 3′ were used for 3′- and 5′-RACE, respectively. RACE PCR was conducted using BD Smart RACE cDNA amplification kit (BD Biosciences Clontech, San Jose, CA) according to the manufacturer's protocol. Subsequently, genespecific primers for *DvvLac2* and *DvvCHS2* were designed to obtain templates for *in vitro* synthesis of dsRNAs. A region of *DvvCHS2* having the greatest sequence divergence between *TcCHS1* and *TcCHS2* was targeted for dsRNA synthesis.

### Design and synthesis of *Diabrotica*-specific dsRNAs

PCR templates for *in vitro* transcription of dsRNAs were generated using the above cDNAs in conjunction with gene-specific primers tailed with the T7 polymerase promoter sequence (TAATACGACTCACTATAGGG). Primers for *DvvLac2* dsRNA synthesis were 5′-(T7)-AACCGCCCACTGTGTACTCAAC-3′ and 5′-(T7)-CACAGCCACCGATCTTCACC-3′, while those for *DvvCHS2* dsRNA synthesis were 5′-(T7)CCCACTGTGTACTCAAC-3′ and 5′-(T7)AGCCACCGATCTTCACC- 3′ which amplify 440 bp and 560 bp fragments, respectively. PCR products were cloned and sequenced. After sequence confirmation, synthesis of long dsRNA was performed using approximately 1 µg of plasmid DNA as template for a 54 µl *in vitro* transcription reaction (MEGAscript® T7 High Yield Transcription Kit, Applied Biosystems/Ambion, www.appliedbiosystems.com). The reaction mix was incubated for four h at 37° C, followed by 15 min of DNase I treatment. The reaction mix was purified using the MEGAclear™ Purification Kit (Applied Biosystems/Ambion) according to the manufacturer's protocol.

### Larval injection

Injection of larvae was performed under a dissecting stereomicroscope. Larvae were anaesthetized by chilling at 4° C for five min, and aligned on double-sided adhesive tape attached to a glass slide to minimize movement during injection. dsRNA was injected using an Eppendorf Transjector 5246 injection system (www.eppendorf.com) with a stainless steel needle holder and borosilicate glass capillary tubes (1.0/0.58 OD/ID; World Precision Instruments, www.wpiinc.com) pulled by a vertical micropipette puller P-30 (Sutter Instruments, http://www.sutter.com). Before their use in microinjections, needles were beveled using a BV-10 micro-pipette beveller (Sutter Instruments) to facilitate exoskeleton penetration.

Serial dilutions of *Lac2* dsRNA were conducted to establish the range at which phenotypic expression would be affected. Dilutions were conducted in two-fold from 200 to 3.12 ng/µl. Approximately 0.2 µl solution was delivered per larva. The doses that generated visible phenotypes were 100– 200 ng/µl, resulting in the delivery of approximately 20–40 ng dsRNA. After the dsRNA concentration optimization step, dsRNA representing each gene was diluted in 0.1 mM sodium phosphate buffer (pH 7, containing 5 mM KC1) to a final concentration of 200 ng/µl. *DvvLac2* dsRNA was injected into second-instar larvae while *DvvCHS2* dsRNA was injected into early third-instars. Control larvae were injected with buffer only. Larvae were injected through the intersegmental membrane of the dorsal abdomen. At least 40 larvae were injected per treatment.

### Phenotypic evaluation of laccase 2 inhibition

Phenotypic evaluation of gene silencing was conducted post-molting. Mature rootworm larvae exhibit highly pigmented and sclerotized anal plates and head capsules, while recently molted larvae lack pigmentation but are fully pigmented within two h after molting (A. Alves, personal observation). Therefore, recently molted third-instars were isolated and aged for 24 h. Larvae with reduced or absent head capsule and anal plate pigmentation were scored as positive for RNAi-mediated gene knockdown. Larvae were flash frozen in liquid nitrogen and RNA isolated for evaluation of transcription level using reverse transcriptase-PCR (RT-PCR) and quantitative RT-PCR (qRT-PCR) (see below).

### RT- and qRT-PCR analysis of transcripts corresponding to DvvLac2 and DvvCHS2

Gene expression among different developmental stages was determined by RTPCR to insure that injections could be timed to occur prior to or during peak expression. Recently molted larvae were identified by lack of pigmentation among those growing on germinated seed corn in transparent gel matrix (Clark et al. 2002). RNA was extracted from third-instars immediately after molt (T_0_), 24 h post-molt (T_1_), and 48 h post-molt (T_2_). Because each instar lasts for approximately seven days under laboratory rearing conditions and because there is no method to determine the age of individual larvae within an instar unless exact time of molt is recorded, RNA was also isolated from randomly selected second- (R_2_) and third- (R_3_) instars and compared with third instars of known ages. Second and third instars were identified based on head capsule width (Ball 1957; George and Hintz 1966). Expression levels for both genes among different instars and ages were determined by RT-PCR (see below).

Post-injection expression levels of *DvvLac2* and *DvvCHS2* were assayed both by RT-PCR and by qRT-PCR. For both RT-PCR and qRTPCR, total RNA was isolated from using TRIzol® Reagent (Invitrogen Life Technologies, Carlsbad, CA) according to the manufacturer's instructions. To assay for *DvvCHS2* silencing, RNA extractions were conducted three days after injection (dai), while the timing of RNA extractions for *DvvLac2* silencing varied depending on when the larvae molted after injection. All larvae involved in *Lac2* experiments were flash frozen in liquid nitrogen 24 h after molting.

For the *DvvLac2* experiment and for comparisons of different developmental stages, RNA was isolated from individual larvae. However, three larvae were pooled for each of the *DvvCHS2* PCR based experiments as described by Arakane et al. (2005b).

Total RNA (1 µg) was used as template for first-strand cDNA synthesis using an oligo(dT) primer. RT-PCR reactions were conducted using the iScript™ cDNA Synthesis Kit (BioRad, www.bio-rad.com) according to vendor's specifications. Controls included a negative reverse transcription reaction that lacked reverse transcriptase to confirm that RNA samples were not contaminated by genomic DNA. qRT-PCR was conducted using SuperScript™ III Platinum® Two-Step RT-PCR Kit with SYBR® Green (Invitrogen Life Technologies, www.invitrogen.com), according to manufacturer's specifications. One microliter of three-fold diluted cDNA was used in each PCR reaction. Primers used in both RT- and qRT-PCR were 5′-TGGGAGAGTGTGGTATCAGACGAG-3′ and 5′-ACACAAATGGCGTCTGCTCTTAC-3′ for *DvvLac2*, which amplified a 172-bp fragment; and 5′TCTTTATTCAGAGCTGGAGCCC-3′ and 5′-CCACCGATCTTCACCTTGAT-3′ for *DwCHS2*, which amplified a 111-bp fragment. The following primers designed for *D. v. virgifera actin* which amplified a 164-bp fragment were used as the internal control for both RT- and qRT-PCR experiments: 5′GTTGGATTCTGGTGATGGTG-3′ and 5′CTCTTTCTGCTGTGGTGGTG-3′. qRTPCR reactions were conducted using the following conditions: 50° C for 2 min incubation, denaturation at 95° C for 2 min followed by 35 cycles of denaturation at 95° C for 30 sec and annealing at 65° C or 60° C for 20 sec for *DvvLac2* and *DvvCHS2*, respectively. RT-PCR reaction products were fractionated by electrophoresis in a 1% agarose gel and visualized by staining with ethidium bromide. qRT-PCR data was analyzed using the standard curve method (Cawthon 2002). Standard amplification curves using the housekeeping gene *actin* in ten-fold serial dilutions were generated to accurately account for differences in amplification efficiency among genes. Standard curves relating the threshold cycle (*Ct*) to the relative concentration of molecules were constructed from five to six data points. The standard curves were used to determine the relative abundance of specific transcripts in each cDNA sample. This was accomplished by comparing the amount of product generated by the templates during exponential amplification. Gene expression was standardized relative to *actin* expression within each sample, and gene expression ratios (dsRNA treated/buffer injected-control) were obtained and established as relative expression values.

### FITC-CBD staining of chitin

Midguts were dissected from actively feeding third instars two days post-injection of *DvvCHS2* dsRNA, or buffer. Guts were stained with a commonly used, high-affinity chitin-binding probe tagged with fluorescein-isothiocyanate (FITC-CBD; New England BioLabs, Inc., www.neb.com) following the method described by Arakane et al. (2005b). Briefly, guts were fixed in 3.7% formaldehyde/PBS (10 mM sodium phosphate buffer, pH 8 containing 100 mM NaCl) for 1 h on ice, followed by three washes with PBS. Midgut tissues (with hindgut still attached) were incubated for 12 h at room temperature with the FITC-CBD probe (1:100 dilution in PBS, pH 8). Excess probe was removed and fluorescence recorded with fluorescent stereomicroscope using light of appropriate excitation and emission wavelengths. Approximately 20 larvae were used for each treatment (dsRNA and control) and each treatment was repeated twice.

## Results

### Homology-based cloning of *D. v. virgifera* lacease 2 and chitin synthase 2

A 1082-bp cDNA was amplified from *D. v. vergifera* using *Lac2*-specific degenerate primers. BlastX searches (Altschul et al. 1997) revealed that the fragment obtained shared 88% and 34% identity with *T. castaneum* Lac2 (TcLac2) and *T. castaneum* Lac1 (TcLac1), respectively (Figure 1). Phylogentic tree analysis of the cDNA using the neighbor-joining method (Saitou and Nei 1987) indicated that the deduced protein of *DvvLac2* forms a single clade with *TcLac2* and not *TcLac1* (phylogeny not shown), Since the cloned fragment shared the most identity with *TcLac2* (Arakane et al. 2005a), this is referred to as *D. v. virgifera* gene as *DvvLac2*. While this cDNA represents only 50% of the gene (aligns with amino acids 241–600 of TcLac2), it still provides sufficient length and specificity to carry out dsRNA-mediated gene silencing.

An 821-bp cDNA was amplified using *CHS2*specific degenerate primers. While BlastX search with the *DvvLac2* sequence as a query (see above) discriminated between *laccase* paralogs, similar analysis of the *DvvCHS* cDNA provided less definitive results. The cloned fragment shared 74% identity with *T. castaneum CHS2* (*TcCHS2*) vs. 69% with *T. castaneum CHS1 (TcCHS1)* (Figure 2). Since the orthology of this *DvvCHS* product was not entirely clear, an additional 864 bp of 5′ sequence and 508 bp of 3′ sequence were obtained using 5′- and 3′ RACE-PCR, respectively. Taken together our *DvvCHS* clones span a total of 2193 bp and the deduced amino-acid sequence shares 65% identity with *TcCHS2*, but only 57% with *TcCHS1*. Phylogentic tree analysis (Saitou and Nie 1987) of the deduced protein indicated that *DvvCHS2* forms a single clade with *TcCHS2*(phylogeny not shown). Therefore, this gene is hereafter referred to as *DvvCHS2*.

Since the above *DvvCHS2* sequence was generated from multiple overlapping clones a single cDNA encompassing the known region was amplified. A 2056-bp amplification product was obtained which corresponds to amino-acid residues 246–923 of *TcCHS2* (data not shown) and provides a *DvvCHS2* cDNA template of sufficient length and specificity to carry out dsRNA-mediated gene silencing.

**Figure 3.**
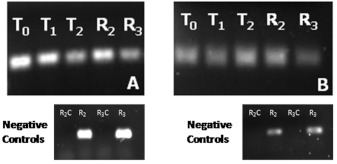
*Diabrotica v. virgifera* gene-expression patterns across different developmental stages based on RT-PCR of (A) *DvvLac2* and (B) *DvvCHS2*. T_0_ = recently molted third-instar larvae, T_1_ = third-instar larvae 24h after molting, T_2_ = third-instar larvae 48h after molting, R_2_ = randomly selected second-instar, and R_3_ = randomly selected third-instar. Negative controls (R_2_C and R_3_C) consisting of PCR reactions lacking reverse transciptase for both *DvvLac2* and *DvvCHS2* were included for randomly selected second- and third-instars to confirm that RNA samples were not contaminated by genomic DNA. High quality figures are available online.

### Expression of laccase 2 and chitin synthase 2

Results from RT-PCT suggest that maximal expression of *DvvLac2* occurs shortly after molting (T_0_) although expression was also detected at both 24 h (T_1_) and 48 h (T_2_), and among randomly selected 2^nd^ and 3^rd^ instars (Figure 3A). A higher level of expression immediately after molting is consistent with the role of *Lac2* in cuticular sclerotization and pigmentation. *DvvCHS2* expression was less variable than *DvvLac2* (Figure 3B) and was detected at approximately equal levels at all stages examined. The relationship of *CHS2* with proper formation of the midgut peritrophic matrix suggests that the enzyme is likely to be expressed in all feeding stages and consistent with the expression pattern observed. In general, both *DvvLac2* and *DvvCHS2* were expressed throughout the larval stages tested. Therefore, the exact timing of dsRNA injection was not critical.

### dsRNA-mediated lacease 2 gene silencing

**Figure 4.**
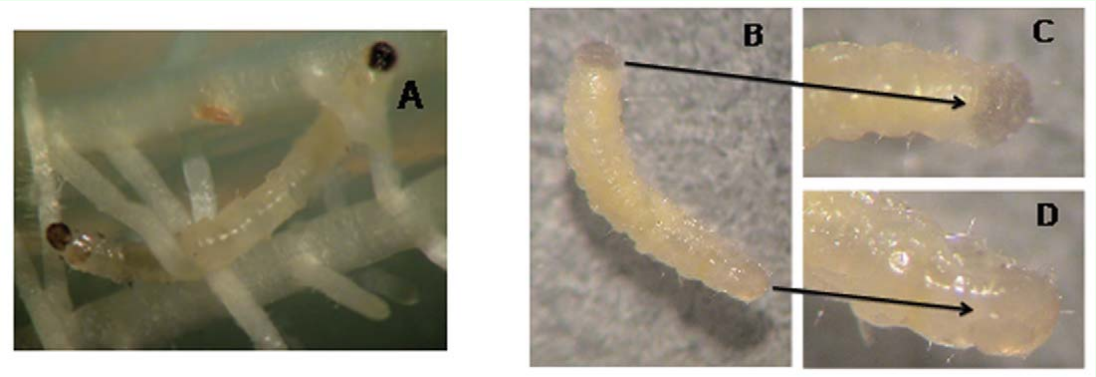
*Diabrotica v. virgifera* larval development and phenotypic expression of *DvvLac2* silencing. (A) Normal pigmentation in a recently molted, buffer-injected larvae 24 h post-molt; (B) *DvvLac2* dsRNA injected larva 24h post-molt; (C) detail of reduced cuticular tanning observed on anal plate and (D) head capsule. High quality figures are available online.

*DvvLac2* dsRNA was injected into secondinstar *D. v. virgifera* to determine the loss-offunction phenotype for *Lac2*. While cuticular tanning of the head capsule and anal plate normally occur within two h after a larval molt (Figure 4A), injection of *DvvLac2* dsRNA interrupted cuticular tanning in 18 of 31 individuals (58%) that were recovered and assayed 24 h after molting (Figure 4B–D). Post-molt cuticular tanning was not affected in any of the 22 buffer-injected larvae recovered (Figure 4A). These results confirm the functional conservation of *Lac2* between *D. v. virgifera* and *T. castaneum*, and demonstrate the efficacy of systemic RNAi in *Diabrotica*.

**Figure 5.**
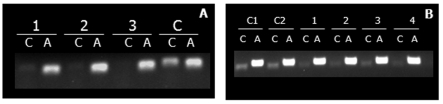
Validation of *laccase 2* (A) and *chitin synthase 2* (B) gene silencing through RNAi in *Diabrotica v. virgifera* larvae injected as second and third instars using RT-PCR. *Laccase 2* dsRNA injected larvae were evaluated 24 h after molting, while *chitin synthase 2* dsRNA injected larvae were evaluated 3 days after injection. The numbers indicate samples injected with dsRNA; C, C1, and C2 are controls (buffer injected samples); L = lacease; A = actin; CS = chitin synthase. High quality figures are available online.

**Figure 6.**
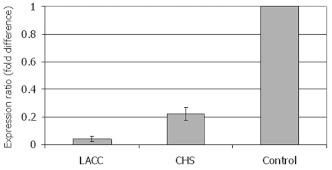
Validation of *DvvLac2* and *DvvCHS2* silencing through RNAi in *Diabrotica v. virgifera* using qRT-PCR. Values are expressed as fold difference of gene expression in the dsRNA injected samples using the buffer injected samples as reference (N = 10). Internal normalization was conducted using *actin* as the housekeeping gene. High quality figures are available online.

Results of RT-PCR show suppression of *DvvLac2* transcript levels in dsRNA-injected larvae compared to controls (Figure 5A). The housekeeping gene *actin* maintained its expression level independent of the treatment (Figure 5A). Inhibition of gene expression averaged 95.8% (Figure 6) relative to control based on qRT-PCR data regardless of whether cuticular tanning was inhibited suggesting that suppression of *DvvLac2* in dsRNA-injected larvae was obtained even when visible phenotype was absent. Mortality resulting from injection trauma was always less than 15% in both control (buffer injected) and experimental (dsRNA injected) groups. Validation was based on both RT-PCR and qRT-PCR, which clearly indicates a reduction in transcript level when comparing dsRNAinjected larvae to control larvae.

### dsRNA-mediated chitin synthase 2 gene silencing

**Figure 7.**
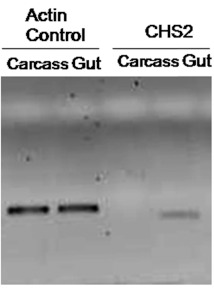
Establishment of exclusive gut specific *DvvCHS2* amplification through RT-PCR (25 cycles) based on RNA extractions from gut and carcass tissues using *DvvCHS2* specific primers. The RT-PCR analysis of rootworm transcripts with the same cDNA templates served as an internal control for normalization of equal sample loading. High quality figures are available online.

Based on the sequence of *DvvCHS2* and differences between *CHS1* and *CHS2* from *T. castaneum*, a set of putative *DvvCHS2*specific primers were designed. The primerpair was tested via RT-PCR using RNA isolated from carcass after removal of gut or from dissected gut tissue extracts as template. Amplification was only observed from gutspecific RNA, suggesting that the primer set was indeed specific for *DvvCHS2* (Figure 7).

Therefore, selectivity of the primer pairs used in the validation of *DvvCHS2* suppression allowed for the exclusive amplification of midgut-specific *CHS2* and not *CHS1* which is expressed in the exoskeleton (Arakane et al. 2005b), and off-target affects on paralogs likely was minimized.

Injection of *DvvCHS2* dsRNA into actively feeding, early third-instar larvae resulted in a substantial decrease in *DvvCHS2* transcript levels when compared to buffer-injected control larvae (Figures 5B, 6). Qualitative assessment of gene silencing obtained by RTPCR showed visual suppression of *DvvCHS2* on dsRNA-injected larvae when compared to the control (Figure 5B). Again, the *actin* gene maintained its expression level independent of the treatment (Figure 5B). Quantitative measurements using qRT-PCR showed an average reduction in *DvvCHS2* transcript level by 78% in dsRNA-injected larvae when compared to the control group (Figure 6).

**Figure 8.**
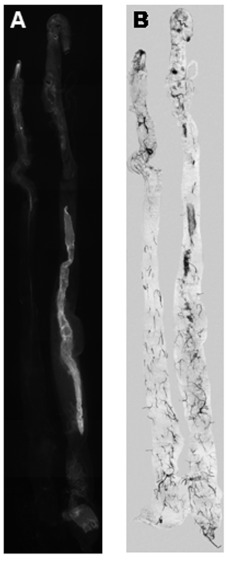
FITC-CBD staining of guts from *DvvCHS2* dsRNA injected and control larvae. The same guts were visualized with both fluorescence (A) and white light (B). Decrease in fluorescence intensity reflects the reduction of chitin synthesis in midgut tissue of *CHS2* dsRNA injected larvae (A, left) compared to control (A, right). High quality figures are available online.

For independent confirmation of reduced gut chitin content in *DvvCHS2* dsRNA-injected larvae, dissected midguts were stained with a fluorescein isothiocyanate-conjugated chitinbinding probe (FITC/CBD). It was assumed that the midgut fluorescence intensity was proportional to the chitin content in the peritrophic membrane. A qualitative decrease in fluorescence intensity was observed in midguts dissected from *DvvCHS2* dsRNAinjected larvae when compared to those from control larvae (Figure 8) suggesting a reduction in chitin content in dsRNA-injected larvae and therefore inhibition of *DvvCHS2*. Approximately 70% of the dsRNA-injected larvae showed visible reduction in fluorescence intensity when compared to control (N=40/treatment). There did not appear to be an adverse effect on larval development in dsRNA-injected larvae, unlike previous observations in *T. castaneum* (Arakane et al. 2005b). However, all injected larvae were processed only three days after injection, and long-term observations of larval development were not conducted.

## Discussion

This study demonstrates that microinjection of long dsRNA (*DvvLac2* or *DvvCHS2*) into *D. v. virgifera* larvae induces gene-specific, systemic, RNAi-mediated silencing of the target transcript as evidenced by both RT- and qRT-PCR (Figures 4–6, 8). Phenotypic effects of gene silencing consisted of visible reduction of cuticular tanning for *DvvLac2*, and reduced chitin staining for *DvvCHS2*. The selective knockdown observed for *DvvCHS2* transcripts suggest that RNAi is a useful tool for silencing midgut-specific genes.

One of the biggest challenges in determining the effectiveness of RNAi in *D. v. virgifera* larvae was being able to observe larval development after injection. The transparent gel matrix used for maintaining corn roots (Clark 2002) was critical to characterizing the phenotypic effects of *Lac2* silencing in *D. v. virgifera*, as it allowed visual observation of developing larvae. However, recovery of intact larvae from the gel medium was difficult, necessitating the development of other rearing methods. Previous attempts to follow larval development in a root/soil mixture or in artificial diet were unsuccessful due to high mortality of injected larvae. However, larval rearing using two-ounce cups with perforated lids and containing peat moss/corn seedling (Laura Campbell, personal communication) with two larvae per cup resulted in survival rates exceeding 60% and enabled successful recovery of larvae after injection.

RNAi-mediated gene silencing in *D. v. virgifera* larvae provides an important tool for investigating alternative pest management approaches. The ability to reliably silence specific genes allows integration of raw genomic information with target discovery and validation, and has been widely recognized as a tool for identifying new drug targets for treating a variety of human diseases (reviewed by Kramer and Cohen 2004). Large-scale gene sequence information is accumulating for a variety of insects, and complete genomic sequence is available for an increasing number of species from a variety of orders. These resources provide a means to conduct comparative sequence analysis for identifying conserved insect genes and to reveal conserved biological functions (McCarter 2004). Such comparative sequence analysis should also allow design of pest management approaches targeting gene products that are unique to pest insects and provide unprecedented selectivity.

Given the specificity of RNAi technology, its use in transgenic plants expressing hairpin RNAs designed to suppress vital genes in specific insect pests upon ingestion offers a new approach to managing crop pests (Baum et al. 2007; Mao et al. 2007). Evidence for selectivity of RNAi transgenic plants expressing *D. v. virgifera* vacuolar *ATPase A* dsRNA is based on the increase in the LC50 of dsRNA by > 10-fold when comparing the effects of rootworm-specific *ATPase A* in *Leptinotarsa decimlineata* with *D. v. virgifera* (Baum et al. 2007). We propose that complete specificity using gene silencing for pest control may be achievable based entirely on nucleotide sequence identity. In this study, this specificity is suggested based on the ability to selectively amplify a single member of a gene family (e.g. chitin synthase 2 and not chitin synthase 1). However, the potential to silence multiple members of a gene family has not been addressed and deserves further investigation.

Development of reliable RNAi techniques for *D. v. virgifera* provides a starting point for assessing gene function and a means to test and validate possible target sites that can be exploited for novel control strategies. Down regulation of *D. v. virgifera* genes by injection of dsRNA observed in the present study as well as delivery by ingestion (Baum et al. 2007) have now been documented for this insect. The possibility of expressing dsRNA constructs specific to *D. v. virgifera* in plants represents a potentially important management option. However, injection of d sRNA provides a means to more accurately deliver specific doses and may be a more efficient delivery method than feeding because larval diet for western corn rootworm is inadequate for more than a few days of larval development (Siegfried et al. 2006). The methods described in this report provide a means to identify and validate potential target sites for developing selective and environmentally safe pest management options and importantly provides a tool for assessing the function of biology and ecology of western corn rootworms.

## Abbreviations

Bt: *Bacillus thuringiensis*
CHS: chitin synthase
dsRNA: double-stranded RNA
Lac: lacease;
RNAi: RNA interference
RT-PCR: reverse transcriptase polymerase chain reaction;
qRT-PCR: quantitative reverse transcriptase polymerase chain reaction
